# Understanding the dynamics of malnutrition dichotomy in India: Trends and insights from the National Family Health Surveys

**DOI:** 10.1016/j.dialog.2025.100209

**Published:** 2025-02-23

**Authors:** Himanshu Jindal, Vinay Suresh, Saniya Agarwal, Priyanshi Vyas, Nabeela Bari

**Affiliations:** a Intern Physician, Ganesh Shankar Vidyarthi Memorial Medical College, Kanpur 208002, India; b Intern Physician, King George's Medical University, Lucknow 226003, India; c Ganesh Shankar Vidyarthi Memorial Medical College, Kanpur 208002, India

**Keywords:** Malnutrition, Undernutrition, Obesity, Nutrition policy, Nutritional status, Dietary diversity

## Abstract

**Purpose:**

India is confronted with a multifaceted malnutrition landscape, characterized by the coexistence of stunting, underweight, and escalating overweight and obesity rates. Current programs predominantly target undernutrition, overlooking the surging prevalence of overweight and obesity. These trends carry substantial economic ramifications, with obesity-related costs expected to rise significantly. Addressing these challenges requires enhanced policy execution and strategic collaboration. This article seeks to help overcome policy inertia in addressing the other end of the malnutrition spectrum—overnutrition.

**Methods:**

The National Family Health Survey (NFHS), a large-scale survey conducted by the Ministry of Health and Family Welfare, Government of India, provides high-quality data on population dynamics and health indicators. Data for relevant health indicators were extracted from NFHS-3 to NFHS-5 to identify trends and shifting paradigms in malnutrition profiles. Data from the latest NFHS (NFHS-5) were briefly analyzed to highlight the malnutrition dichotomy and perform linear regression analysis.

**Results:**

The data revealed a declining trend in stunting and underweight prevalence alongside a rise in overweight prevalence. Linear regression analyses on NFHS-5 data showed a positive association between literacy and obesity in both men and women. The findings also indicated that children fed with minimum dietary diversity were more likely to be overweight, and revealed a significant association between elevated random blood glucose levels and obesity in women.

**Conclusions:**

By implementing the necessary interventions and strategies, India can establish a holistic approach to addressing both undernutrition and overnutrition effectively, thus contributing to Sustainable Development Goal-2 and paving the way for a healthier and more productive future for India's population.

## Highlights

• Undernutrition and obesity coexist as dual burdens in India's malnutrition landscape, complicating policy response.
• NFHS data reveal that high literacy is linked to obesity and dietary diversity impacts child health.
• Rising obesity and persistent undernutrition require balanced, targeted interventions and multi-sectoral policy reforms.

## 1 Introduction

India ranks 105th out of 127 countries in the 2024 Global Hunger Index. However, undernutrition is not the only problem faced in terms of the nutritional status of the population. Obesity is a growing concern, especially in the age group of 15–49 years, with 24 % of women and 22.9 % of men in India being overweight or obese (NFHS-5). Adding to this problem is the fact that nutrition programs in India primarily address undernutrition, posing challenges in addressing overnutrition [1].

In India, the trends in malnutrition indicators have witnessed a paradigm shift [2]. While undernutrition is the major problem in children, overnutrition prevalence is high in adults. Despite the decline in malnutrition rates from 1990 to 2022, India faces a dual threat of malnutrition, with high rates of both underweight and obesity [3].

This article aims to shift focus to India's malnutrition spectrum by analyzing National Family Health Surveys' (NFHSs) data to highlight contrasting trends in undernutrition and overnutrition. It explores key socioeconomic, dietary, and demographic factors driving these trends and underscores regional disparities in malnutrition prevalence. By doing so, we hope to help overcome policy inertia and inform evidence-based targeted interventions for addressing the multifaceted challenge of malnutrition and contributing to sustainable nutrition security in India.

## 2 Materials and methods

The National Family Health Surveys (NFHSs), conducted by the Ministry of Health and Family Welfare, Government of India, provide nationally representative, cross-sectional data on demographic, health, and nutrition indicators. The data analyzed in this study were extracted from publicly available NFHS datasets, focusing on nutrition related indicators in both children and adults. Descriptive statistics were employed to summarize trends in undernutrition and overnutrition indicators as they offer a straightforward approach to identifying key patterns and variability within the data. Geographic disparities in malnutrition indicators were mapped using state-level data to visualize the distribution of underweight and overweight prevalence in both children and adults.

A brief linear regression analysis was performed on NFHS-5 data to explore the association between specific socioeconomic and dietary variables and the risk of overweight or obesity. Linear regression was chosen for its ability to model relationships between continuous and categorical variables, enabling an understanding of the potential linear relationship between variables while controlling for other covariates.

## 3 Results

**Fig. 1:**
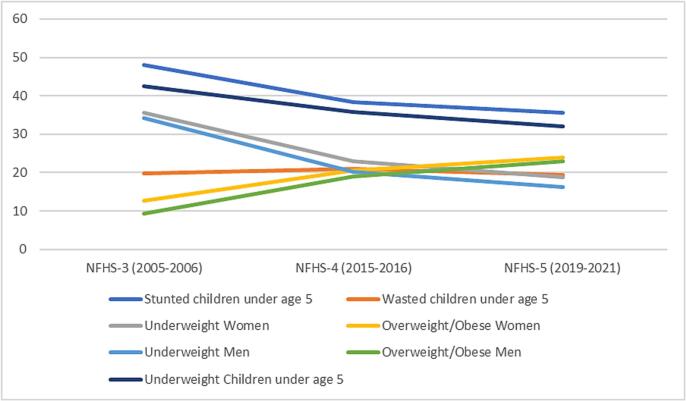
Trends in malnutrition indicators in India (NFHS-3 to NFHS-5). This figure illustrates the trends in various malnutrition indicators from the National Family Health Surveys (NFHS-3: 2005–2006, NFHS-4: 2015–2016, and NFHS-5: 2019–2021). Overnutrition (overweight/obese) prevalence has nearly doubled in both genders, rising from 12.6 % to 24 % in women and from 9.3 % to 22.9 % in men between NFHS-3 and NFHS-5 while underweight prevalence has almost halved in both genders, declining from 35.6 % to 18.7 % in women and from 34.2 % to 16.2 % in men during the same period. Undernutrition indicators in children under age 5 show declining trends but the decrease is modest.

The NFHS (NFHS-3: 2005–06; NFHS-5: 2019–21) data revealed a significant decline in stunting prevalence among children under five years of age, dropping from 48 % in NFHS-3 to 35.5 % in NFHS-5. Similarly, underweight prevalence decreased from 42.5 % to 32.1 % during the same period. Conversely, overweight prevalence has risen sharply; among adults aged 15–49 years, the percentage of overweight or obese women increased from 12.6 % in NFHS-3 to 24 % in NFHS-5, while men showed an increase from 9.3 % to 22.9 % (Fig. 1).

**Fig. 2:**
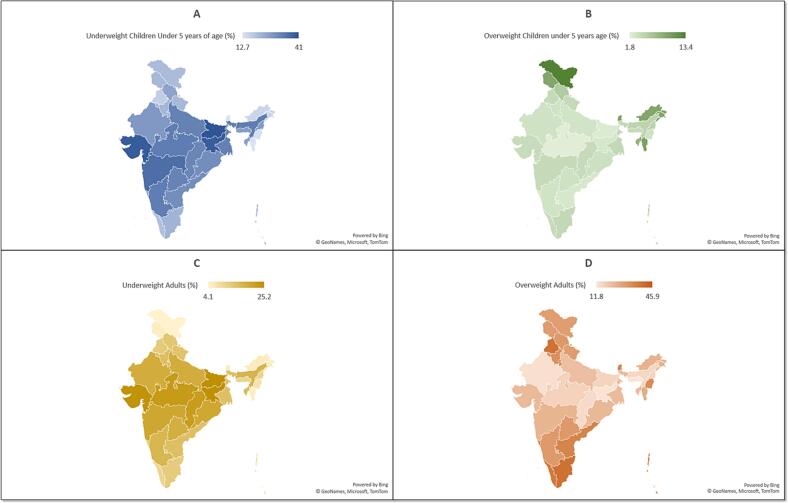
Heatmaps representing the percentage of underweight and overweight individuals across different states in India, based on the NFHS-5 survey data. A. Prevalence of underweight children under 5 years of age, with the highest rates observed in central and eastern states. B. Prevalence of overweight children under 5 years of age, which is relatively low across most states, but higher in northern and northeastern regions. C. Prevalence of underweight adults, predominantly affecting states in central and southern India. D. Prevalence of overweight adults, which is most pronounced in urbanized and southern states, demonstrating a rising trend of overnutrition. Data source: International Institute for Population Sciences (IIPS) and ICF. 2021. National Family Health Survey (NFHS-5), 2019–21: India. Mumbai: IIPS. Refer to supplementary data sheet 1.

**Fig. 3:**
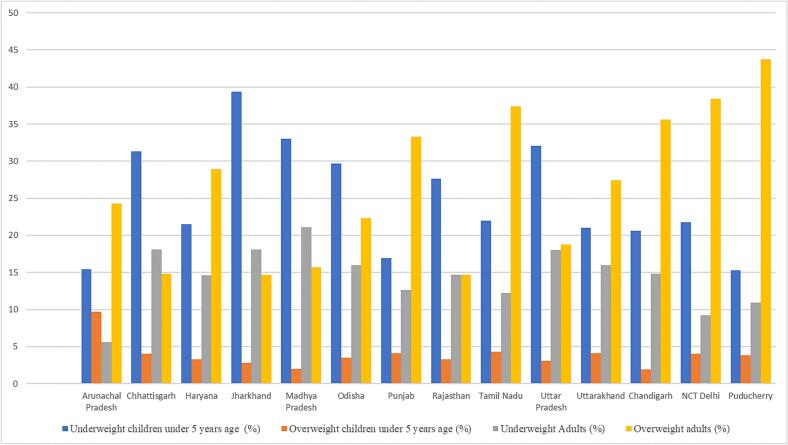
Malnutrition dichotomy in major Indian states and union territories. This figure illustrates the percentage of underweight children under 5 years of age (blue), overweight children under 5 years of age (orange), underweight adults (gray), and overweight adults (yellow) across various Indian states and union territories. Regions such as Central and South India, including Delhi, Chandigarh, Puducherry, and Tamil Nadu, exhibit notably high overweight prevalence in adults. Conversely, Jharkhand has the highest prevalence of underweight children under 5 years of age. Other states, including Madhya Pradesh, Uttar Pradesh, and Chhattisgarh, follow with elevated rates of underweight children in that order. Data source: International Institute for Population Sciences (IIPS) and ICF. 2021. National Family Health Survey (NFHS-5), 2019–21: India. Mumbai: IIPS. Refer to supplementary data sheet 2.

Historically, the most widespread forms of malnutrition in India have been stunting, wasting, and micronutrient deficiencies. Although recent trends, as per NFHS reports, suggest improvements in these health indicators over the past decades, progress has been uneven and inequitable. Meanwhile, the prevalence of obesity in India is rising faster than the world average [4]. This paradox underscores the urgent need to address both ends of the malnutrition spectrum. State-level data extracted from the NFHS-5 (Supplementary Data Sheet 1) were used to generate geographic heat maps illustrating the significant regional variations in underweight and overweight prevalence across Indian states (Fig. 2). Fig. 3 highlights the malnutrition dichotomy across major Indian states and Union Territories (Supplementary Data Sheet 2). These patterns underscore the need for region-specific interventions to address malnutrition effectively.

Linear regression analyses identified several critical associations:

• The percentage of children aged 6–23 months fed Minimum Dietary Diversity (MDD) was significantly associated with overweight prevalence in children under 5 years of age (*t* = 2.227, *p* = 0.033).
• The percentage of literate men (*t* = 2.826, *p* = 0.022) and literate women (*t* = 3.246, *p* = 0.012) was significantly associated with the prevalence of overweight or obesity.
• The percentage of women who are overweight or obese (BMI ≥25.0 kg/m^2^) was significantly associated with very high (>160 mg/dl) random blood glucose levels (*t* = 2.740, *p* = 0.025).

## 4 Discussion

WHO defines malnutrition as nutrient intake imbalances, including undernutrition and obesity. NFHS-5 data show 35.5 % of Indian children under five are stunted, 19.3 % wasted, and 32.1 % underweight. Severe anemia impacts 57 % of Indian women. Malnutrition rates are higher in rural areas than in urban areas, driven primarily by socioeconomic disparities, maternal education, age, and nutritional status, low birth weight, and religious variations [5].

The WHO describes overweight and obesity as an unhealthy or excessive buildup of body fat. For adults, BMIs over 25 and 30 are classified as overweight and obese, respectively. For children <5 years old, having a weight-for-height measurement of more than 2SD is classified as overweight, and obese as having a weight-for-height measurement exceeding 3SD. A linear regression model based on NFHS-5 data identified a positive association between literacy and obesity in both men and women, although this relationship is likely confounded by socioeconomic status. Socioeconomic factors correlate with both higher literacy rates and increased obesity prevalence, suggesting that the observed relationship may not be directly causal. Additionally, elevated random blood glucose levels (>160 mg/dl) were significantly associated with obesity (BMI ≥25.0 kg/m^2^) in women, likely reflecting glucose intolerance driven by adiposity. This finding aligns with a multivariate analysis of NFHSs' data which links rising overweight/obesity rates to higher socioeconomic status (“better-off families”), smoking, unclean household cooking fuels, and diabetes [6, p. 5227]. In 2019, noncommunicable diseases linked to a higher-than-optimal BMI caused approximately 5 million deaths [7]. If no action is taken, the global economic burden of overweight and obesity is projected to escalate to $3 trillion annually by 2030 [8].

NFHS-5 data reveal that only 11.3 % of children aged 6–23 months were fed the minimum acceptable diet, leaving over 88 % susceptible to micronutrient deficiencies and potential metabolic issues, such as increased waist-to-hip ratios, later in life [9,10]. This challenge is exacerbated by the processed food industry, which promotes calorie-dense yet nutrient-poor products, compounding the risk of malnutrition [2,11].

### 4.1 Implications

Malnutrition dichotomy in India poses significant health risks, particularly across vulnerable age groups. The physical effects of undernutrition can lead to lifelong health challenges, including weakened immune systems and greater susceptibility to infections [12]. Overnutrition, meanwhile, is contributing to rising rates of overweight/obesity and non-communicable diseases. This places a dual strain on healthcare systems.

The psychological effects of malnutrition, especially among children and adolescents cannot be neglected. Malnourished children may experience poor cognitive skills, productivity losses, early morbidity and mortality, and reduced educational attainments, perpetuating cycles of poverty [13]. Overnutrition, coupled with social stigma, can lead to mental health issues like anxiety, depression, and body image disorders [14]. While the economic losses can be quantified, the psychological distress and social stigma faced by the affected individuals are also significant. This creates a need for more focused and coordinated programs.

It also hinders the government from developing effective policies, programs, and infrastructure, as conflicting priorities and resource allocation challenges arise. While the existing policies disproportionately focus on undernutrition, it is vitally important to address obesity as well [15]. As of 2019, the cost of obesity in India is estimated to be $23.24 billion with $5.13 billion attributed to direct medical costs. It is estimated to grow 19 folds by 2060 if the current trends continue [8]. This is paradoxical in a country like India which is home to one-third of the world's malnourished population as per the 2018 Global Nutrition Report. Women and children, particularly in rural and economically disadvantaged communities, face the brunt. Pregnant women with inadequate nutrition risk complications, including low birth weight and maternal mortality [16]. Children under five from impoverished households are most at risk of stunting, wasting, and developmental issues (weakened immunity, reduced IQ, etc.) [17,18]. Targeted interventions could reduce malnutrition, potentially raising India's GDP by 3 %. [19]. The health effects of malnutrition have led to rapidly rising non-communicable diseases, translating into higher medical expenses [20]. According to the National Health Accounts Estimates for India 2019–2020 report, the out-of-pocket healthcare expenditure has reached up to 47.07 % in 2019–2020.

### 4.2 Potential solutions and recommendations

The challenge extends beyond policy creation to effective implementation, which can be strengthened through capacity building, accountability, and community engagement. For instance, community health workers can play a pivotal role in monitoring and promoting nutritional programs at the grassroots level [21,22]. Community-based approaches are often more sustainable and culturally more appropriate. Engaging local leaders and volunteers can enhance the reach and impact of nutritional programs [22,23]. While large-scale, structured community-based nutrition models are still developing in India, localized initiatives have shown promise. A study supported by 10.13039/100006641UNICEF and conducted by Vitamin Angels India observed significant improvements in dietary diversity and nutritional outcomes in communities with strong local leadership and grassroots engagement [24]. Effectively addressing both ends of the malnutrition spectrum necessitates a multi-sectoral collaboration as demonstrated by successful models from countries such as Brazil, Peru, and Burkina Faso (West Africa) [21,[25], [26], [27]].

#### 4.2.1 Role of food fortification

The United Nations' Sustainable Development Goal 2 (SDG-2) aims to eradicate hunger and ensure food security and improved nutrition by 2030. The Food Fortification Resource Centre of FSSAI highlights that India faces a significant burden of micronutrient deficiencies, leading to widespread “hidden hunger” among its population. Fortification is essential for combating micronutrient deficiencies. Although schemes for fortifying salt, cereals, milk, and edible oils are already in place, stronger government mandates and enforcement for specific food categories are needed to ensure widespread adoption by mass producers. Public-private partnerships and awareness campaigns can help drive the success of fortification initiatives. Food fortification is a standard approach for micronutrient deficiencies, yet nutrition science increasingly supports dietary diversification for sustainable, long-term impact [28,29]. The EAT-Lancet commission recommended diet for rural India costs approximately $3.00- $5.00 per person per day, while the current average dietary intake cost is only about $1.00 per person per day [30]. It is financially difficult for many to access sustainable diets. Food fortification provides a cost-effective solution to address nutritional deficiencies, ensuring good-quality staple foods reach a broader population. As overnutrition—driven by high-calorie intake, poor dietary quality, and unsustainable eating patterns—continues to rise, food fortification can help address this issue by enriching staple foods with essential nutrients and potentially curbing reliance on ultra-processed products [16,31].

#### 4.2.2 Role of dietary diversity

Dietary diversity, the consumption of a range of food groups, is vital to promoting health and food security, providing macro- and micronutrients for growth and development. Diversified diets are essential for maintaining overall health by providing a balanced range of nutrients. However, less than 1 % to 7 % of the population in India consume foods with adequate dietary diversity, which is still richer in rural than in urban areas [32,33]. NFHS-5 data shows that in most Indian states, under 50 % of children aged 6–23 months receive adequate dietary diversity, with Meghalaya and Sikkim as exceptions where this percentage is 54.2 % and 54 %, respectively. Uttar Pradesh ranks lowest at 14.1 %. Data on the minimum acceptable diet show similar trends, with only 5.9 % of children in Uttar Pradesh meeting criteria compared to 28.5 % and 23.8 % in Meghalaya and Sikkim. Interestingly, linear regression analysis of the data revealed that children fed with minimum dietary diversity were more likely to be overweight.

Urbanization and lifestyle changes have led to the decline of traditional diets, which are typically diverse and healthy. Dietary diversity is shaped by landscape heterogeneity, socioeconomic status, and livelihood [34]. Strengthening the production and consumption of diverse, locally adapted, and nutrient-dense foods can critically enhance dietary diversity in India [35]. In India, urban households with higher income and education levels exhibit greater dietary diversity. Wild foods and traditional food systems can promote healthy, sustainable diets, especially in resource-poor areas [[36], [37], [38], [39]]. Individual dietary diversity scores (DDS) show that women and children often have lower diversity compared to men, highlighting intra-household food distribution disparities [40]. A study conducted in Iran found that participants with obesity had a higher DDS compared to those who were overweight or of normal weight [41]. In contrast, a study in Tanzania reported inconsistent positive correlations between DDS and the prevalence of overweight [42]. Meanwhile, a systematic review and meta-analysis found no significant association between DDS and BMI status, highlighting the need for further research on this topic [43]. Household food insecurity and dietary diversity are linked to undernutrition in children under five, while diversified diets from an early life can protect children from malnutrition [44].

To enhance the effectiveness of interventions, policy frameworks should prioritize strengthening dietary diversity through locally adapted, nutrient-rich food systems while reinforcing the role of community-based nutrition programs. Integrating traditional food knowledge with modern dietary guidelines can improve dietary habits sustainably.

#### 4.2.3 Role of food hygiene

In Southeast Asia, almost 50 % of malnutrition is associated with inadequate water and sanitation facilities, as well as unhygienic practices that result in fatal diseases [45]. NFHS-5 data reveal that diarrhea, a common foodborne illness, affected 222,234 children between 2019 and 2021, highlighting gaps in food hygiene and sanitation. According to WHO estimates, while 10 % of foodborne illnesses are reported in developed countries, only 1 % are documented in developing nations. Similarly, in India, the true burden of foodborne illnesses remains largely unknown, as only cases with high mortality or those occurring in urban areas are more likely to have been reported. The Integrated Disease Surveillance Program in India reported 2688 foodborne disease outbreaks between 2010 and 2018, leading to 153,745 illnesses and 572 deaths primarily due to contaminated grains and beans, with the highest number of deaths resulting from chemical contamination [46]. Raising awareness about food spoilage, recognizing foodborne illness symptoms, and enforcing stringent food safety regulations are crucial steps. Despite the significant socioeconomic impact of foodborne illnesses, there are few effective measures to mitigate them, largely due to the lack of a comprehensive surveillance system [47].

## 5 Conclusion

To address India's malnutrition dichotomy, it is crucial to prioritize interventions targeting both undernutrition and overnutrition. Community-based approaches, leveraging the role of local health workers, can enhance program monitoring and implementation at the grassroots level. Strengthening dietary diversity through locally adapted and nutrient-dense food systems, coupled with large-scale food fortification initiatives, can mitigate nutritional deficiencies sustainably. Policymakers should prioritize funding for studies to explore causative factors, including the impact of urbanization, processed food consumption, and lifestyle changes on nutritional status. Policies should integrate evidence-based insights from studies exploring the socio-economic and behavioral factors influencing malnutrition trends. Furthermore, implementing strict food safety regulations, and fostering multisectoral collaborations across health, education, and agriculture sectors will be pivotal in achieving long-term nutrition security. These efforts must be complemented by capacity-building and accountability frameworks to ensure the effective execution of the strategies.

Promoting healthy food choices and ensuring access to suitable food supplies are crucial. As the UN's food security report states: “Future generations will only thrive as productive actors and leading forces in food systems if decisive action is taken to ensure that children are no longer deprived of their right to nutrition”. Active community participation in nutritional interventions is needed. By incorporating the necessary recommendations, India can develop a roadmap for tackling malnutrition, addressing both undernutrition and obesity and contributing to the global goal of ending hunger and malnutrition by 2030.

## CRediT authorship contribution statement

**Himanshu Jindal:** Writing – review & editing, Writing – original draft, Validation, Supervision, Formal analysis, Data curation, Conceptualization. **Vinay Suresh:** Writing – review & editing, Writing – original draft, Methodology, Formal analysis. **Saniya Agarwal:** Writing – original draft, Validation, Data curation. **Priyanshi Vyas:** Writing – original draft, Validation, Data curation. **Nabeela Bari:** Writing – original draft, Data curation.

## Declaration of competing interest

The authors declare that they have no competing interests.

## Appendix A Supplementary data

State-wise nutritional and health indicators from NFHS-5, including underweight and overweight prevalence among children and adults, BMI distribution, hypertension levels, blood glucose levels, and substance use patterns. Supplementary material 1

Summary of underweight and overweight prevalence data among children and adults across major Indian states and union territories based on NFHS-5 data. Supplementary material 2
